# Characterization of a novel automated microfiltration device for the efficient isolation and analysis of circulating tumor cells from clinical blood samples

**DOI:** 10.1038/s41598-020-63672-7

**Published:** 2020-05-05

**Authors:** Juan F. Yee-de León, Brenda Soto-García, Diana Aráiz-Hernández, Jesús Rolando Delgado-Balderas, Miguel Esparza, Carlos Aguilar-Avelar, J. D. Wong-Campos, Franco Chacón, José Y. López-Hernández, A. Mauricio González-Treviño, José R. Yee-de León, Jorge L. Zamora-Mendoza, Mario M. Alvarez, Grissel Trujillo-de Santiago, Lauro S. Gómez-Guerra, Celia N. Sánchez-Domínguez, Liza P. Velarde-Calvillo, Alejandro Abarca-Blanco

**Affiliations:** 1 Delee Corp., Mountain View, CA 94041 USA; 2 Departamento de Bioquímica y Medicina Molecular, Facultad de Medicina, Universidad Autónoma de Nuevo León, Monterrey, 64460 Mexico; 3 Department of Chemistry and Chemical Biology, Harvard University, Cambridge, MA 02138 USA; 4 Centro de Biotecnología-FEMSA, Escuela de Ingeniería y Ciencias, Tecnologico de Monterrey, Monterrey, 64849 Mexico; 5 Departamento de Bioingeniería, Escuela de Ingeniería y Ciencias, Tecnologico de Monterrey, Monterrey, 64849 Mexico; 6 Departamento de Mecatrónica e Ingeniería Eléctrica, Escuela de Ingeniería y Ciencias, Tecnologico de Monterrey, Monterrey, 64849 Mexico; 7 Servicio de Urología, Hospital Universitario “Dr. José Eleuterio González”, Universidad Autónoma de Nuevo León, Monterrey, 64460 Mexico

**Keywords:** Urological cancer, Biomedical engineering, Biomarkers

## Abstract

The detection and analysis of circulating tumor cells (CTCs) may enable a broad range of cancer-related applications, including the identification of acquired drug resistance during treatments. However, the non-scalable fabrication, prolonged sample processing times, and the lack of automation, associated with most of the technologies developed to isolate these rare cells, have impeded their transition into the clinical practice. This work describes a novel membrane-based microfiltration device comprised of a fully automated sample processing unit and a machine-vision-enabled imaging system that allows the efficient isolation and rapid analysis of CTCs from blood. The device performance was characterized using four prostate cancer cell lines, including PC-3, VCaP, DU-145, and LNCaP, obtaining high assay reproducibility and capture efficiencies greater than 93% after processing 7.5 mL blood samples spiked with 100 cancer cells. Cancer cells remained viable after filtration due to the minimal shear stress exerted over cells during the procedure, while the identification of cancer cells by immunostaining was not affected by the number of non-specific events captured on the membrane. We were also able to identify the androgen receptor (AR) point mutation T878A from 7.5 mL blood samples spiked with 50 LNCaP cells using RT-PCR and Sanger sequencing. Finally, CTCs were detected in 8 out of 8 samples from patients diagnosed with metastatic prostate cancer (mean ± SEM = 21 ± 2.957 CTCs/mL, median = 21 CTCs/mL), demonstrating the potential clinical utility of this device.

## Introduction

In the last two decades, circulating tumor cells (CTCs) have attracted a significant amount of attention for their potential use as a blood-based biomarker for a broad range of cancer-related clinical applications. CTCs are malignant cells that are shed from the primary and/or metastatic solid tumors and then infiltrate into the vascular and lymphatic systems; these cells play a fundamental role in the metastatic process of non-hematological cancers^1–3^. Although the first report describing the existence of CTCs dates from 1869^4^, the heterogeneity and the extremely low concentration of these cells in regard to the cellular components of blood, about 1–10 CTCs per 10^9^ blood cells, makes their capture extremely challenging^5,6^. It was not until the recent development of technologies with the required sensitivity and reproducibility, that the possibility to perform CTC-based clinical assays started to become a reality.

To date, numerous studies have shown that CTCs can be used as a prognostic indicator of disease progression and overall survival in patients with metastatic breast, prostate, and colorectal cancer^7–10^. In addition, changes in the CTC burden in patients over time have been associated with the effectiveness of the administered therapies^11–13^. Furthermore, the phenotypic and genotypic analysis of CTCs can enable the continuous assessment of mutations that confer therapeutic sensitivity or resistance to targeted therapies, providing information that is of paramount importance for cancer treatment personalization^14–16^. Recent studies suggest that CTCs may even have the potential to be used as a biomarker for recurrence and early cancer detection^11,17,18^.

Most of the technologies developed to isolate CTCs from blood are based on sample enrichment methods that depend on specific antigen-antibody interactions, such as microfluidic devices functionalized with biomolecules that act as targeting ligands^19–21^ or platforms that use micro- or nano-magnetic particles coated with specific antibodies as a mean to isolate these rare cells^22–24^. Although these technologies have demonstrated clinical utility, a fundamental problem of these approaches is the lack of a universal surface marker that is consistently expressed by CTCs. Most of these technologies, including the CellSearch system, which is considered the current gold standard, use EpCAM (epithelial cell adhesion molecule) antibodies to selectively trap cancer cells to the functionalized substrate/particles^25^. However, CTCs intravasate into the bloodstream by undergoing a process known as the epithelial-mesenchymal transition (EMT), in which their epithelial phenotype is downregulated, including the expression of EpCAM antigens. This fact limits the capture of CTC subpopulations with diminished expression of this specific surface marker, thereby losing valuable information^26,27^. Consequently, there exists a need for technologies with different capture approaches that are independent of surface markers expressed by CTCs.

An effective alternative to these technologies are microfiltration devices, which rely on the differences in size and deformability between blood cells and CTCs in order to capture them. These platforms have consistently proven their effectiveness at isolating a greater number of CTCs in samples from patients with different types of cancer, even capturing CTC subtypes that no longer express EpCAM antigens, when compared with approaches based on capture antibodies^28–30^.

Various microfiltration technologies such as the commercially available ISET^31^ and ScreenCell^32^ devices, use polycarbonate track-etch membranes to isolate CTCs from blood. However, the processes used to fabricate these membranes create random pores in the plastic sheet, making them inadequate for this end. The porosity of these membranes cannot exceed from 2% without overlapping between pores, while increasing the number of pores beyond this point can hinder the separation efficiency of cells with similar size in regard to the pore size^33^. The membranes are therefore restricted to a low porosity, which can lead to cell damage (due to uneven distribution of pressure over the membrane), lower capture efficiency, reduced reproducibility, and a higher recovery of non-specific cells (which can potentially clog the membrane)^34,35^.

To circumvent these issues, other approaches have utilized membranes crafted through microfabrication techniques. The advantage of these fabrication processes is the capacity to produce membranes with uniform patterns, where the size, geometry, quantity, and distribution of pores can be precisely controlled; allowing higher porosities and thereby mitigating the issues aforementioned. To capture CTCs, several groups have employed microfabricated membranes made from different materials, such as parylene C^34,36,37^, SU-8^35,38^, and silicon^39,40^. However, the fabrication processes for these membranes are complex, making their production costly and hardly scalable, limiting their introduction into clinical practice. Recently, photolithography-based electroforming, a technique that enables the production of highly precise metal structures, has been utilized to fabricate filters used for the isolation of cellular subpopulations, including CTCs from blood samples^41–43^. In contrast with the ones created with other manufacturing methods, these membranes could be mass produced at a significantly lower cost.

Nevertheless, despite the type of membrane used, the microfiltration technologies developed so far are not capable of processing, preparing, and analyzing the captured cells in an automated way^28,29,31,32,34–41,43^; easily leading to human error and cell loss due to the manual steps that must be performed. Additionally, performing a proper enumeration of the captured CTCs requires optical analysis with highly specialized microscopes that may not be available in all clinical settings.

In order to standardize the use of CTCs as a biomarker in clinical practice, it is fundamental to acknowledge the necessity of developing platforms with high recovery rates and the capacity of carrying out the entire sample workflow, without human intervention. In this manuscript, we present a novel automated microfiltration device that utilizes electroformed nickel membranes and a machine-vision-enabled microscope specifically designed for the efficient isolation and analysis of CTCs. Its performance was characterized using blood samples from healthy donors spiked with cells from prostate cancer cell lines. Furthermore, the results obtained after processing samples from patients with diagnosed metastatic prostate cancer and healthy male donors are also discussed in this work.

## Methods

### Fabrication of membranes

**Figure 1:**
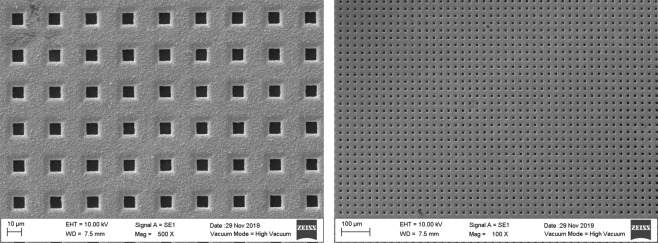
SEM micrographs of a 7 *μ*m pore size membrane, taken at 500X (left) and 100X (right) magnifications.

Nickel membranes were fabricated using conventional lithography and electroplating techniques, following the methodology described by Warkiani *et al*.^44^, and characterized by scanning electron microscopy (SEM) to verify their dimensions and homogeneity, as shown in Fig. 1.

### Holder fabrication

**Figure 2:**
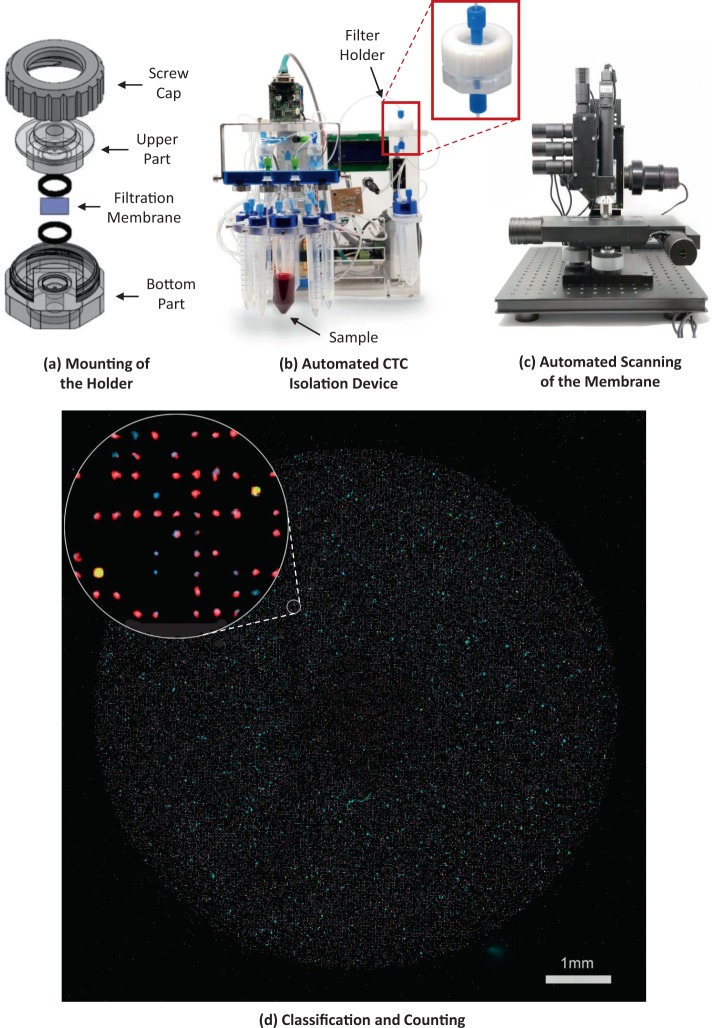
Sample processing and analysis workflow. (**a**) Schematic showing the parts that composed the holder where the membrane is placed. (**b**) A blood sample is automatically processed through the microfiltration device, followed by immunostaining steps. The membrane is mounted on a microscope slide and images are acquired using the integrated imaging system (**c**). (**d**) Fluorescent events are automatically classified and counted by a machine-vision algorithm implemented in the imaging system. Scale bar: 1 mm.

To prevent leakage during sample processing, each membrane was placed on a custom-made PMMA holder fabricated with a computer numerical control (CNC) micro-milling machine (MDA Precision, Morgan Hill, California, USA). This holder is constituted by an upper part, a bottom part, and a screw cap, as shown in Fig. 2a. The upper and bottom parts have a 1/4″- 28 threaded flat-bottom port that allows an easy connection to external components using standard microfluidic fittings. Both parts, also have a 0.9 mm microchannel that disembogue into a microchamber of 130 *μ*L, designed to minimize the holder's dead volume and ensure an appropriate distribution of flow throughout the processing area. The membrane is sandwiched between the upper and bottom parts of the holder along with two O-rings, and the setup is fastened by screwing in the cap to the thread milled in the holder's bottom part. The sample processing area, which is the surface of the membrane that is in contact with the sample, is delimited by the holder and has a 9 mm diameter. This translates to an approximate number of 110440, 101790, and 94110 pores for the 7, 8, and 9 *μ*m pore size membranes, respectively, corresponding to a porosity of 8.50%, 10.24%, and 11.98% each.

### Blood extraction

Blood samples from healthy male donors and patients with diagnosed metastatic prostate cancer were provided by the urology service of the “Dr. José Eleuterio González” University Hospital, according to the protocol approved by their Institutional Review Board with number UR16-0007. Prior to blood extraction, informed consent was obtained from healthy donors and cancer patients. Samples were collected in 6 mL BD Vacutainer K_2_EDTA blood collection tubes (BD, Franklin Lakes, New Jersey, USA) and were processed by our device within 3 hours after the extraction. All procedures involving human participants were performed in accordance with the 1964 Helsinki declaration and its later amendments or comparable ethical standards.

### Cell culture of cancer cell lines

Human prostate cancer cell lines (ATCC, Manassas, Virginia, USA), PC-3 (CRL-1435), LNCaP clone FGC (CRL-1740), DU-145 (HTB-81), and VCaP (CRL-2876) were cultured using F-12K, RPMI-1640, EMEM, and DMEM media (ATCC, Manassas, Virginia, USA), respectively. All media were supplemented with 10% fetal bovine serum (FBS) (ATCC, Manassas, Virginia, USA). Cultures were incubated at 37 °C in an air atmosphere of 5% CO_2_ and harvested from T-25 flasks using 0.25% trypsin - 0.02% EDTA solution (Thermo Fisher Scientific, Waltham, Massachusetts, USA) when 80% confluence was reached. Cells were quantified with a hemocytometer and viability was assessed by trypan blue dye exclusion. A viability of at least 95% was achieved after harvesting.

### Preparation and processing of spiked blood samples for capture efficiency and purity assessment

To evaluate the device performance, PC-3, LNCaP, DU-145, or VCaP prostate cancer cells were pre-stained prior to being spiked into blood samples from healthy donors by incubating cell cultures with 25 *μ*M of CellTracker orange CMRA (Thermo Fisher Scientific, Waltham, Massachusetts, USA) for 45 min at 37 °C in an air atmosphere of 5% CO_2_. Cells were harvested using 0.25% trypsin - 0.02% EDTA solution (Thermo Fisher Scientific, Waltham, Massachusetts, USA) and counted with a hemocytometer. The resulting cell suspension was serially diluted to achieve a concentration of approximately 100 cancer cells per 30 *μ*L; that volume was then added to the blood samples. Subsequently, spiked blood samples were diluted with a 0.3% formaldehyde - 0.15% pluronic F68 solution in PBS to a 1:2 v/v ratio and incubated for 10 min at room temperature before being processed by the device, at either 2 or 3 mL/min flow rates, using membranes with a pore size of 7, 8, or 9 *μ*m. Once the samples were filtered, 0.1% pluronic F68 solution in PBS was flowed to wash out blood cells remaining on the membrane, followed by fixation and nuclear staining, which were carried out by incubating the membrane for 10 min after passing 1 mL of 4% formaldehyde and 1 *μ*g/mL Hoechst 33342 solutions at 500 *μ*L/min, respectively. At the end of each incubation, 0.1% pluronic F68 solution in PBS was used to wash the remnants of the fixative and nuclear staining dye. Finally, the holder was disassembled and the membrane was mounted on a microscope slide using Fluoromount-G (Thermo Fisher Scientific, Waltham, Massachusetts, USA) for its subsequent analysis by fluorescence microscopy.

To properly estimate the number of tumor cells spiked into blood samples, equal volumes of the cancer cell suspension were added to 10 wells of a 96-well plate. The capture efficiency of the microfiltration device was determined by comparing the number of cancer cells trapped on the membrane against the average number of cells counted on the wells. To calculate purity, the total number of tumor cells was divided by the total number of nucleated events along the membrane.

### Preparation and processing of spiked blood samples for viability assessment

The LIVE/DEAD assay was used to evaluate cell viability. PC-3 cancer cells were pre-stained with CellTracker blue CMF_2_HC (Thermo Fisher Scientific, Waltham, Massachusetts, USA) and spiked into samples from healthy donors at a concentration of 1000 cells per 7.5 mL of blood. Spiked samples were diluted with a 0.15% pluronic F68 solution in PBS to a 1:2 v/v ratio and incubated for 10 min at room temperature before being processed by the device. Once the samples were filtered, 0.1% pluronic F68 solution in PBS was flowed at 500 *μ*L/min during 10 min to wash out blood cells unspecifically captured on the membrane. Afterward, 1 mL of 2 *μ*M calcein AM - 4 *μ*M ethidium homodimer-1 solution was passed at 500 *μ*L/min, followed by a 45 min incubation. Then, the holder was disassembled and the membrane was mounted on a microscope slide for its analysis by fluorescence microscopy.

Blue/green fluorescent events were classified as live tumor cells, whereas blue/red events were counted as dead cancer cells. Cell viability was calculated as a percentage by dividing the total number of live tumor cells by the total number of cancer cells along the membrane.

### Preparation and processing of cancer cell suspensions for the evaluation of cells’ clonogenic potential

To assess the clonogenic potential of cancer cells after filtration, cell suspensions, consisting of 1000 PC-3 cells spiked into 7.5 mL of 0.15% pluronic F68 solution in PBS, were processed by the device at a flow rate of 2 mL/min. Prior to processing, 70% ethanol was flowed through the system for 30 min to ensure sterility. Once filtered, membranes were detached from the holder and gently rinsed with F-12K media supplemented with 10% FBS and 2X antibiotic-antimicotic solution (Thermo Fisher Scientific, Waltham, Massachusetts, USA) to transfer the captured cells into the wells of a 12-well plate. Recovered cells were incubated at 37 °C in an air atmosphere of 5% CO_2_ for a total of 8 days to evaluate if they were still able to proliferate.

### RT-PCR analysis and sequencing

Blood samples from healthy donors, spiked with 15, 50, 250, 500, and 1000 LNCaP cells, were processed by the device at a flow rate of 2 mL/min. After filtration, 0.1% pluronic F68 solution in PBS was flowed at 500 *μ*L/min for 10 min to wash out blood cells unspecifically captured on the membranes before transferring them to microcentrifuge tubes for nucleic acid extraction. Total RNA was isolated from the captured cells using the AllPrep DNA/RNA FFPE kit (Qiagen, Venlo, Netherlands), according to the manufacturer’s instructions, excluding the deparaffinization steps. Full-length cDNA was produced from total RNA by first-strand cDNA synthesis using the SuperScript IV First-Strand Synthesis System (Thermo Fisher Scientific, Waltham, Massachusetts, USA), following the manufacturer's instructions. A 599 base pair coding region of the ligand binding domain (LBD) of the androgen receptor (AR) was amplified using the Platinum SuperFi PCR Master Mix (Thermo Fisher Scientific, Waltham, Massachusetts, USA) and the following primer pairs: forward 5′-CCAATGTCAACTCCAGGATGCTCTAC-3′, and reverse 5′-AATTCCCCAAGGCACTGCAGA-3′. PCR amplicons were purified using the PureLink Quick Gel Extraction kit, for further Sanger sequencing. Sequencing electropherograms were compared to the reference sequence of the AR (NM000044.4) to identify the missense mutation T878A.

### Preparation, processing, and on-membrane immunofluorescence staining of blood samples from healthy donors and patients with metastatic prostate cancer

Blood samples of 7.5 mL from healthy donors and patients diagnosed with metastatic prostate cancer were diluted with a 0.3% formaldehyde - 0.15% pluronic F68 solution in PBS to a 1:2 v/v ratio and incubated for 10 min at room temperature before being processed through the device at a flow rate of 2 mL/min. Once the samples were filtered, 0.1% pluronic F68 solution in PBS was flowed to wash out blood cells unspecifically captured on the membrane. Fixation was performed by flowing 1 mL of 4% formaldehyde in PBS at 500 *μ*L/min, followed by a 10 min incubation. Subsequently, permeabilization was carried out by passing 1 mL of 0.3% PBST at 500 *μ*L/min, followed by a 10 min incubation. Afterward, to prevent non-specific binding of antibodies, blocking was made by flowing 1 mL of 1% BSA - 0.1% PBST solution at 500 *μ*L/min, followed by an incubation of 30 min. Then, 500 *μ*L of an antibody cocktail containing 8 *μ*g/mL of alexa fluor 488 labeled anti-cytokeratin (pan reactive) (clone C-11), alexa fluor 647 labeled anti-human CD45 (clone HI30), and biotin labeled anti-human PSMA (FOLH1) (clone LNI-17) antibodies (BioLegend, San Diego, California, USA) in 1% BSA - 0.1% PBST solution was flowed through at 250 *μ*L/min, followed by a 1 hour incubation. Finally, 500 *μ*L of a mixture containing 1 *μ*g/mL of Hoechst 33342 and 8 *μ*g/mL of streptavidin-alexa fluor 568 conjugate (Thermo Fisher Scientific, Waltham, Massachusetts, USA) in 1% BSA - 0.1% PBST solution was flowed at 250 *μ*L/min, followed by a 1 hour incubation. Washing steps of 5 min were carried out at 500 *μ*L/min after finishing each incubation with the solutions described above. After fixation, and for the final washing step, 0.1% pluronic F68 in PBS was used, while 0.1% PBST solution was utilized in between to wash out the residues of the remaining solutions.

### Statistical analysis

All the experiments with prostate cancer cell lines were performed in triplicates. These results, along with the ones obtained in clinical samples, are reported as mean ± standard error of the mean (SEM). The difference between means was determined by using the unpaired t-test, where a p-value less than 0.05 was considered statistically significant.

## Results

### Automated microfiltration device and imaging system

We built an automated microfiltration device and an imaging system for the efficient isolation and rapid analysis of CTCs from blood samples. Nickel membranes with square-shaped pores of 7, 8, and 9 *μ*m size were employed for processing blood samples; all of them with a 17 *μ*m pore spacing.

A custom-made holder was fabricated to secure membranes and prevent leakage during filtration. Once assembled, the holder is connected to a fully automated flow control unit (Fig. 2b), which consists of a diaphragm compressor as pressure source, an electronic proportional valve that controls the pressure applied to the sample and reagents reservoirs, an electronic rotary valve to select from which reservoir liquid is expelled, and a flow sensor that provides the necessary feedback to precisely compute the pressure needed in the selected reservoir to maintain the desired flow rate. The entire system is commanded by a microcontroller that follows user-defined protocols, which can be programmed using a graphical user interface in a personal computer. This platform is able to process blood samples and perform on-filter fixation and immunostaining of the captured cells automatically, without requiring disassembly of the holder.

After sample processing, the membrane is mounted on a microscope slide and images of the entire membrane are acquired with our imaging system comprised of a four-channel fluorescence microscope with a motorized stage, an autofocus routine that allows the scanning of the membrane with high precision (Fig. 2c), and a machine vision algorithm that automatically counts the fluorescent events categorized as CTCs (Fig. 2d). Moreover, further corroboration of classified cells can be performed by a qualified technician through a software interface that allows the individual visualization of cells that were classified as CTCs. In this study, cells categorized as CTCs were automatically classified by the algorithm implemented in the imaging system, these events were further corroborated by a trained operator. A detailed description and validation of the imaging system performance can be found in Aguilar-Avelar *et al*.^45^. In addition, if molecular analysis are required, the membrane can be collected in a microtube for nucleic acid extraction using commercially available kits.

The diagram depicted in Supplementary Fig. S1 shows the workflows that were followed to process and analyze patients’ and control samples, as well as the spiked blood samples used to demonstrate the molecular analysis feasibility.

### Capture efficiency and purity assessment

To characterize the performance of our microfiltration device, PC-3 cancer cells were pre-stained with CellTracker orange CMRA and spiked into blood samples from healthy donors at a concentration of 100 cells per 7.5 mL. The samples were diluted, prefixed, and processed by the device, followed by on-membrane fixation and nuclear staining, as described in the Methods section. Subsequently, an image of the entire membrane was acquired with our imaging system, and fluorescent events were automatically classified and enumerated.

Capture efficiency and purity were evaluated using membranes with pore sizes of 7, 8, and 9 *μ*m and flow rates of 2 and 3 mL/min. Irrespective of the membrane and flow rate tested, spiked blood samples were processed without seeing any signs of coagulation.

**Figure 3:**
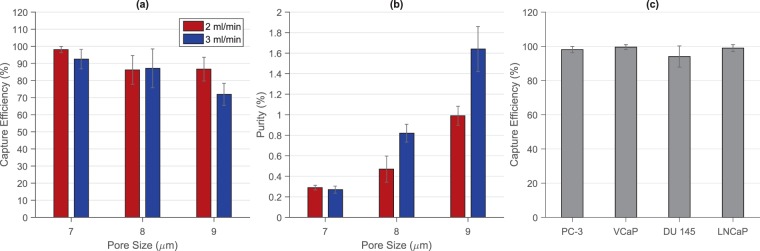
(**a**) Capture efficiencies and (**b**) purities obtained after filtering 7.5 mL blood samples spiked with 100 PC-3 cancer cells using membranes with pore sizes of 7, 8, and 9 *μ*m at flow rates of 2 and 3 mL/min. (**c**) Capture efficiencies obtained after filtering 7.5 mL blood samples spiked with 100 PC-3, VCaP, DU-145, and LNCaP cancer cells using membranes with a pore size of 7 *μ*m at a flow rate of 2 mL/min. Error bars represent the standard error of the mean (*n* = 3).

The membranes pore size and the flow rate at which the samples were processed, demonstrated to be directly related to the capture efficiency of the device, as depicted in Fig. 3a. When utilizing membranes with a pore size of 7 *μ*m and processing samples at a flow rate of 2 mL/min, an average capture efficiency of 98.09% ± 1.82% was obtained, dropping to 93% ± 5.69% when the flow rate was increased to 3 mL/min. When samples were processed through membranes with a pore size of 8 *μ*m, the capture efficiency was maintained above 85%, regardless of the flow rate established. Similar values were observed while using membranes with a pore size of 9 *μ*m and filtering samples at 2 mL/min, plummeting to 72% ± 6.45% when the flow rate was set to 3 mL/min.

Overall, the rate of contaminant events decreased as the pore size of the membranes was increased and samples were processed at higher flow rates. An average of 745 ± 65 nucleated events per mL were captured when samples were filtered through membranes of 9 *μ*m pore size under flow rates of 3 mL/min, in comparison to the 4144 ± 315 nucleated cells per mL captured when using membranes of 7 *μ*m pore size and samples were processed at flow rates of 2 mL/min. Considering the number of tumor cells captured after processing spiked samples, these results corresponded to an average purity of 1.64% ± 0.22% and 0.29% ± 0.02%, respectively, as shown in Fig. 3b.

**Figure 4:**
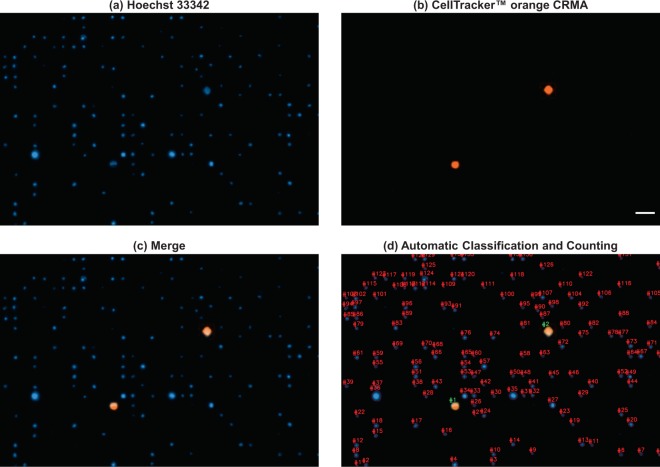
Representative micrograph of cells captured after filtering a 7.5 mL blood sample spiked with 100 pre-stained PC-3 cells; (**a**) Hoechst 33342, (**b**) CellTracker orange CMRA, and (**c**) Merge. (**d**) Automated classification and counting of fluorescent events. Scale bar: 50 *μ*m.

Based on the high capture efficiency obtained and despite the number of recovered non-specific events, the following experiments were carried out with membranes of 7 *μ*m pore size and samples being processed at a flow rate of 2 mL/min. Figure 4 shows a representative micrograph of the captured cells after processing a spiked blood sample under this set of parameters, as well as the results obtained after performing the automatic classification and counting.

### Capture efficiency using different prostate cancer cell lines

Besides the characterization made with PC-3 cells, the performance of the microfiltration device was also assessed using the VCaP, DU-145, and LNCaP prostate cancer cell lines. Similarly, 7.5 mL blood samples from healthy donors were spiked with 100 pre-stained cells and processed through the device as described in the Methods section. Capture efficiencies of 99.51% ± 1.47%, 94% ± 3.60%, and 98.92% ± 2.06% were obtained for VCaP, DU-145, and LNCaP, respectively, as seen in Fig. 3c. The number of contaminant events was maintained at similar levels to those described for the PC-3 cell line experiments.

The relative standard deviation (RSD), obtained from the experiments carried out with the different cell lines, ranged between 1.93 and 6.65%. Altogether, the results suggest that our microfiltration device has a high capture efficiency and assay reproducibility when processing spiked samples.

### Viability assessment

The LIVE/DEAD assay was used to evaluate cell viability. To identify tumor cells from the background of blood cells captured in the membrane, PC-3 cells were pre-stained with CellTracker blue CMF_2_HC before being spiked into blood samples from healthy donors. Tumor cell viability was calculated by quantifying the number of blue/green (viable) and blue/red (dead) fluorescent events along the membrane. An average viability of 90.3% ± 0.88% was achieved after filtering spiked blood samples, in comparison to the 96% ± 1.53% observed in the initial tumor cell suspensions. Furthermore, based on their ability to grow into colonies, we have validated that captured cells maintain their clonogenic potential after filtering, if samples are not previously prefixed, as depicted in Supplementary Fig. S2. These results demonstrate that the shear stress exerted on cells during processing is minimal, enabling high recovery rates of viable cells if desired.

### Molecular analysis

**Figure 5:**
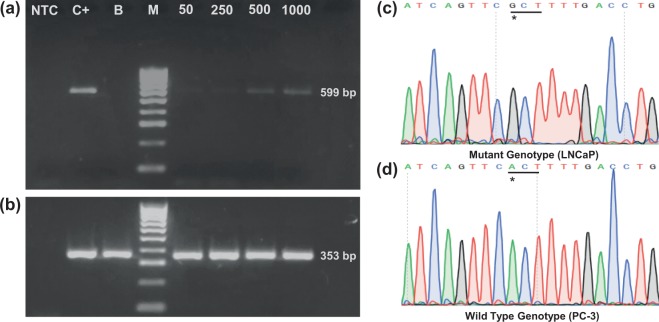
(**a**) RT-PCR analysis of the AR transcript. NTC: Negative control; C+: Positive control; B: Non-spiked blood sample; M: Molecular weight marker; 50: Blood sample spiked with 50 LNCaP cells; 250: Blood sample spiked with 250 LNCaP cells; 500: Blood sample spiked with 500 LNCaP cells; 1000: Blood sample spiked with 1000 LNCaP cells. (**b**) ACTB was used as a control to assess cDNA synthesis. Images of the DNA stained gels were acquired using an exposure time of 330 ms and cropped to provide clarity, uncropped images were included in Supplementary Fig. S3. The AR point mutation T878A was identified by comparing the electropherogram obtained after processing a blood sample spiked with 50 LNCaP cells (**c**) with the one acquired from a suspension containing only PC-3 cells (**d**). The underline denotes the nucleotides comprising the AR 878 codon and the asterisks indicate the nucleotide that is switched.

To demonstrate that our device is compatible with standard molecular analysis, 15, 50, 250, 500, and 1000 LNCaP cells were spiked into 7.5 mL blood samples from healthy donors and processed through the device for the identification of AR transcripts by RT-PCR. AR gene expression was consistently confirmed by agarose gel electrophoresis in samples spiked with as low as 50 LNCaP cells (approximately 7 cells per mL), while non-spiked blood samples resulted negative, as shown in Fig. 5a,b. In addition, the presence of the AR point mutation T878A, harbored by the LNCaP cell line, was successfully identified by Sanger sequencing after processing a blood sample spiked with 50 LNCaP cells and comparing the results with the ones derived from analyzing a sample containing only PC-3 cells, which possess the wild-type genotype, as depicted in Fig. 5c,d.

The PCR products obtained from the membranes used to filter samples spiked with 15 LNCaP cells (approximately 2 cells per mL) were successfully amplified by hemi-nested PCR (hn-PCR), enabling the detection of AR transcripts in two of the three experiments done with this concentration. This suggests that a lower limit of detection could be achievable with the use of more sensitive techniques, such as qRT-PCR or digital PCR (dPCR). No DNA bands were seen after reamplification of PCR reactions obtained from the membranes used to filter non-spiked blood samples.

### Detection of CTCs from samples of patients with metastatic prostate cancer

To demonstrate that our platform can isolate CTCs from clinical samples, we used our device to process 8 samples of patients diagnosed with metastatic prostate cancer (age range: 56 to 80 years), who were under a therapeutic regimen, and 8 samples from healthy male donors (age range: 30 to 54 years), followed by on-membrane immunostaining as described in the protocol detailed in the Methods section. Cells were categorized as CTCs based on the clinically valid definition of CTC, which is a nucleated cell larger than 4 *μ*m that expresses epithelial proteins, such as cytokeratins 8, 18, and/or 19, while being negative for the leukocyte-specific antigen CD-45^46^. Moreover, we have incorporated the expression of the prostate specific membrane antigen (PSMA) as a parameter to categorize cells as CTCs; PSMA is a transmembrane protein that is usually overexpressed in nearly all malignant prostate cancer cells and its expression increases as the disease progresses^47,48^. The categorization of CTCs based on PSMA expression is sustained by studies that report the successful isolation of CTCs from blood samples of prostate cancer patients using antibodies against PSMA as the capture strategy^21,49,50^. Therefore, in this work, nucleated cells with the phenotypes CK+/PSMA+/CD45-, CK+/PSMA-/CD45-, and CK-/PSMA+/CD45- were enumerated as CTCs.

**Figure 6:**
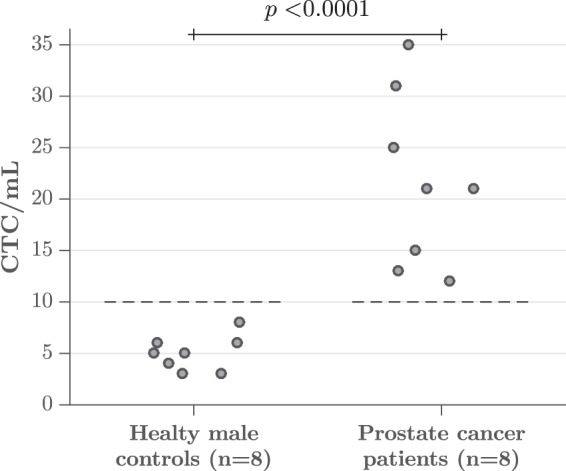
CTC count obtained after processing blood samples from 8 healthy male donors and 8 metastatic prostate cancer patients. The number of events categorized as CTCs in control samples led us to establish a threshold of clinical significance of 10 CTCs/mL. The differences between the number of CTCs found in these two groups were statistically significant (*p* < 0.0001).

The number of cells categorized as CTCs, after analyzing control samples, ranged from 3 to 8 cells per mL (mean ± SEM = 5 ± 0.597 CTCs/mL, median = 5 CTCs/mL). These results led us to establish a threshold of clinical significance of 10 CTCs/mL. The CTC count in all samples from patients surpassed this value, ranging from 12 to 35 cells per mL (mean ± SEM = 21 ± 2.957 CTCs/mL, median = 21 CTCs/mL). The difference between the means of both groups was statistically significant (*p* < 0.0001). Figure 6 summarizes the results obtained after the analysis of patients’ and control samples.

**Figure 7:**
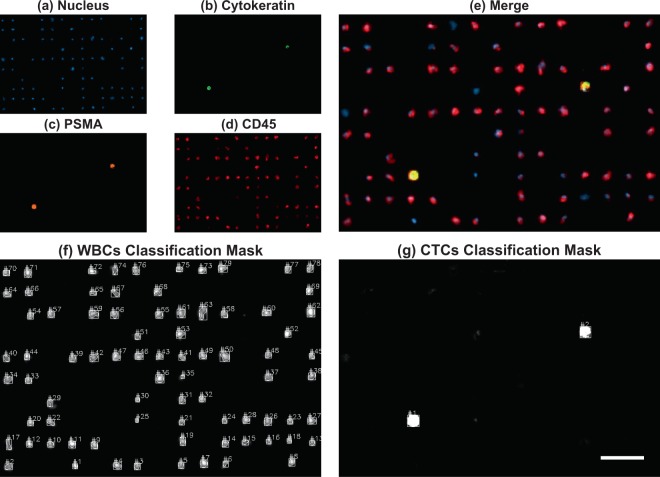
Fluorescent micrograph of CTCs isolated from a blood sample of a patient with metastatic prostate cancer; (**a**) Nucleus, (**b**) Cytokeratin, (**c**) PSMA, (**d**) CD45, and (**e**) Merge. Classification masks generated for the identification of (**f**) WBCs and (**g**) CTCs. Scale bar: 50 *μ*m.

An average of 6235 ± 321 leukocytes per mL were captured after processing patients’ samples, in comparison to the 4916 ± 429 contaminating cells per mL enumerated when control samples were filtered. The elevation of these cells may result from the fact that some patients with solid tumors will develop a paraneoplastic leukemoid reaction^51,52^, increasing the number of white blood cells. Considering the number of CTCs and leukocytes enumerated after processing patients’ samples, an average purity of 0.37% ± 0.048% was calculated. Figure 7 shows a micrograph of CTCs isolated from a patient diagnosed with metastatic prostate cancer, as well as the resulting classification masks generated by the counting software.

Nucleated cells with a CK+/PSMA+/CD45+ phenotype were also observed in patients’ and control samples. The isolation of cells expressing epithelial, tissue-specific, and leukocyte markers has been reported in many instances and with different technologies^11,53^. Interestingly, these events were found in greater numbers than the cells classified as tumoral, and were more prevalent in patients’ samples (mean ± SEM = 85 ± 24 events/mL, median = 59 events/mL) than in controls (mean ± SEM = 15 ± 2 events/mL, median = 13 events/mL), however, they were not counted as CTCs.

## Discussion

CTCs have demonstrated their potential as a blood-based biomarker that can be used on a broad range of cancer-related clinical applications. This has been possible due to the recent development of several technologies, which have enabled the isolation and subsequent analysis of these malignant cells. However, despite the capture approach used, the non-scalable fabrication, prolonged sample processing times, and the lack of automation, associated to most of these technologies, represent substantial limitations that impede their transition from research tools to solutions employed in the standard clinical practice.

In this manuscript, we presented a novel automated microfiltration device that integrates an imaging system for the efficient isolation and rapid analysis of CTCs from blood samples. Due to the rarity of CTCs, technologies able to rapidly process high volumes of sample are desirable. Our platform can process 7.5 mL blood samples in less than 12 minutes, in comparison with other technologies that can only process significantly lower amounts of sample and/or require hours to do so^12,19,54^. On the other hand, our device makes possible automatic on-membrane fixation and immunostaining without needing to disassemble the holder, enhancing practicality and avoiding cell loss during the staining process.

The integrated imaging system comprises a four-channel fluorescence microscope with a motorized stage and an autofocus routine that was specifically designed for scanning the entire membrane with high precision. The relevance of the above relies on the fact that usually the filters suffer a deformation while processing samples, resulting fundamental for the microscope to be capable of focusing automatically when scanning large areas. Moreover, it also possesses a machine-vision algorithm that automatically counts the fluorescent events categorized as CTCs, thereby eliminating the subjective interpretation of operators and increasing the reproducibility of analysis, while decreasing the time needed to manually enumerate the CTCs captured. Further corroboration of classified cells can also be performed by a trained technician through a software interface that allows the individual visualization of cells selected as CTCs. These attributes facilitate the identification of predictive and therapeutic markers that can be expressed by CTCs, such as AR-V7, HER-2, EGFR, among others.

High capture efficiencies were achieved after processing spiked blood samples from healthy donors. Four different prostate cancer cell lines were used to characterize the device performance, and it was found that the capture efficiency of this platform was greater than 93% among all the cell lines tested, having small coefficients of variation, which denotes high reproducibility. Our recovery rates were superior than the ones obtained by several microfiltration devices^36–38,40,55,56^. In addition, it was observed that the level of cellular contamination did not affect the identification of cells by immunostaining. Moreover, viable tumor cells were recovered after processing unfixed samples, demonstrating that the shear stress exerted on cells during filtering did not compromise their integrity. The processing of unfixed blood samples spiked with PC-3 cancer cells resulted in a significant reduction in the number of leukocytes captured (from 4144 ± 315 to 345 ± 17 cells/mL), while the capture efficiency remained unchanged. However, sample prefixation was performed because the literature extensively reports that CTCs could be smaller than cells from cancer cell lines^20,57^. Although processing prefixed samples elevates the number of the contaminating events captured, it also increases the chances of capturing small CTCs. Therefore, in this manuscript, the characterization of the device performance was reported using prefixed samples.

We were also able to identify the AR point mutation T878A from 7.5 mL blood samples spiked with 50 LNCaP cells (approximately 7 cells per mL) using RT-PCR and Sanger sequencing, demonstrating that the captured cells remain suitable for further molecular analysis. The relevance of this finding is that missense mutations often lead to the development of therapeutic resistance in several types of cancer. The noninvasive identification of these point mutations can aid physicians to monitor changes in tumor genotypes during the course of treatments, enabling the personalization of cancer therapies. Furthermore, the detection of AR transcripts from 7.5 mL blood samples spiked with 15 LNCaP cells (approximately 2 cells per mL) by hn-PCR, suggests that the implementation of more sensitive techniques aimed at enriching target molecules, such as qRT-PCR and dPCR, could increase our limit of detection. Besides, due to the easy detachment of the membrane from the holder, single cells could be isolated by micropipette aspiration or by attaching a micromanipulator to the imaging system to perform single cell sequencing in order to enable the study of CTCs heterogeneity in cancer patients, unlike other filter-based microdevices that require additional steps to retrieve the tumor cells captured, which leads to further cell loss^38,58^.

In this work, blood samples from 8 patients diagnosed with prostate cancer were processed and analyzed, as well as 8 control samples from healthy donors. Events classified as CTCs were observed in control samples, leading us to establish a threshold of clinical significance of 10 CTCs per mL, similar to that reported by Stott *et al*.^20^. CTCs above this threshold were detected in all patients’ samples, and their number was higher than those observed in control samples, even though some of these patients were clinically responding to the administered therapies. The number of CTCs captured by our device, points to the feasibility of monitoring the dynamic changes in CTC burden over time, implying that the treatment effectiveness follow-up would also be possible with our system.

Over 80% of the CTCs found in patients’ samples presented dual expression of cytokeratin and PSMA. Variability in the cytokeratin staining intensity, which is reported to occur during the EMT, was observed in CTCs, while PSMA expression remained consistent, supporting the use of this marker to discriminate between CTCs and blood cells in patients with advanced prostate cancer. Moreover, nucleated cells with a CK+/PSMA+/CD45+ phenotype were also observed in both the patients’ and control samples. These cells were found in a greater concentration than CTCs, and appear with a higher frequency in the patients’ samples than in controls; these findings were similar to those reported when using other technologies^11,53^. Nevertheless, the clinical significance of these events warrants further study.

In conclusion, CTCs have demonstrated their potential as a powerful biomarker that can be continuously assessed to determine phenotypic and genotypic changes that confer therapeutic sensitivity/resistance during the course of cancer treatments. However, most of the technologies designed to capture these rare cells are not easily transferable to clinical practice. We have developed a novel membrane-based microfiltration device that integrates a fully automated sample processing unit and a machine-vision-enabled imaging system for the efficient isolation and rapid analysis of CTCs from blood; the platform allows the automation of the sample processing, immunostaining steps, analysis, and classification of fluorescent events for the identification of cancer cells. Capture efficiencies greater than 93% and coefficients of variation below 7% were achieved after processing samples from healthy donors spiked with different prostate cancer cell lines, while the isolated cells remained viable and suitable for molecular analysis. Moreover, CTCs above the established threshold of 10 cells per mL were detected in every sample from the 8 patients with metastatic disease. Although large-scale clinical studies still need to be done, these results, in addition to the fast processing time and large sample volume that can be processed by our platform, make this device a promising tool that can be rapidly integrated into clinical practice. Currently, a study with a larger cohort of prostate cancer patients is being carried out in order to continue the validation of this technology and the assessment of its clinical potential.

## Supplementary information

Supplementary Information.
