# Supplementary material for: Characterization of a novel automated microfiltration device for the efficient isolation and analysis of circulating tumor cells from clinical blood samples
Source: Sci Rep. 2020 May 5;10:7543. doi: 10.1038/s41598-020-63672-7 (PMC7200708; doi:10.1038/s41598-020-63672-7)

Characterization of a novel automated microfiltration device for the efficient isolation and analysis of circulating tumor cells from clinical blood samples - Supplementary information

Juan F. Yee-de León[1,*], Brenda Soto-García[1,*], Diana Aráiz-Hernández[1,*], Jesús Rolando Delgado-Balderas[1,2,*], Miguel Esparza[1], Carlos Aguilar-Avelar[1], J. D. Wong-Campos[1,3], Franco Chacón[1], José Y. López-Hernández[1], A. Mauricio González-Treviño[1], José R. Yee-de León[1], Jorge L. Zamora-Mendoza[1], Mario M. Alvarez[4,5], Grissel Trujillo-de Santiago[4,6], Lauro S. Gómez-Guerra[7], Celia N. Sánchez-Domínguez[2], Liza P. Velarde-Calvillo[1,+], and Alejandro Abarca-Blanco[1,+]

1 Delee Corp., Mountain View, CA, 94041, USA.
2 Departamento de Bioquímica y Medicina Molecular, Facultad de Medicina, Universidad Autónoma de Nuevo León, Monterrey, 64460, Mexico.
3 Department of Chemistry and Chemical Biology, Harvard University, Cambridge, MA, 02138, USA.
4 Centro de Biotecnología-FEMSA, Escuela de Ingeniería y Ciencias, Tecnologico de Monterrey, Monterrey, 64849, Mexico.
5 Departamento de Bioingeniería, Escuela de Ingeniería y Ciencias, Tecnologico de Monterrey, Monterrey, 64849, Mexico.
6 Departamento de Mecatrónica e Ingeniería Eléctrica, Escuela de Ingeniería y Ciencias, Tecnologico de Monterrey, Monterrey, 64849, Mexico.
7 Servicio de Urología, Hospital Universitario "Dr. José Eleuterio González", Universidad Autónoma de Nuevo León, Monterrey, 64460, Mexico.
+ Correspondence and requests for materials should be addressed to L.P.V.C. (liza@delee.bio) or A.A.B. (alejandro@delee.bio).
* These authors contributed equally to this work.

Supplementary Fig. S1 – This diagram shows the workflows that were followed to process and analyze patients' and control samples, as well as the spiked samples used to demonstrate the molecular analysis feasibility.

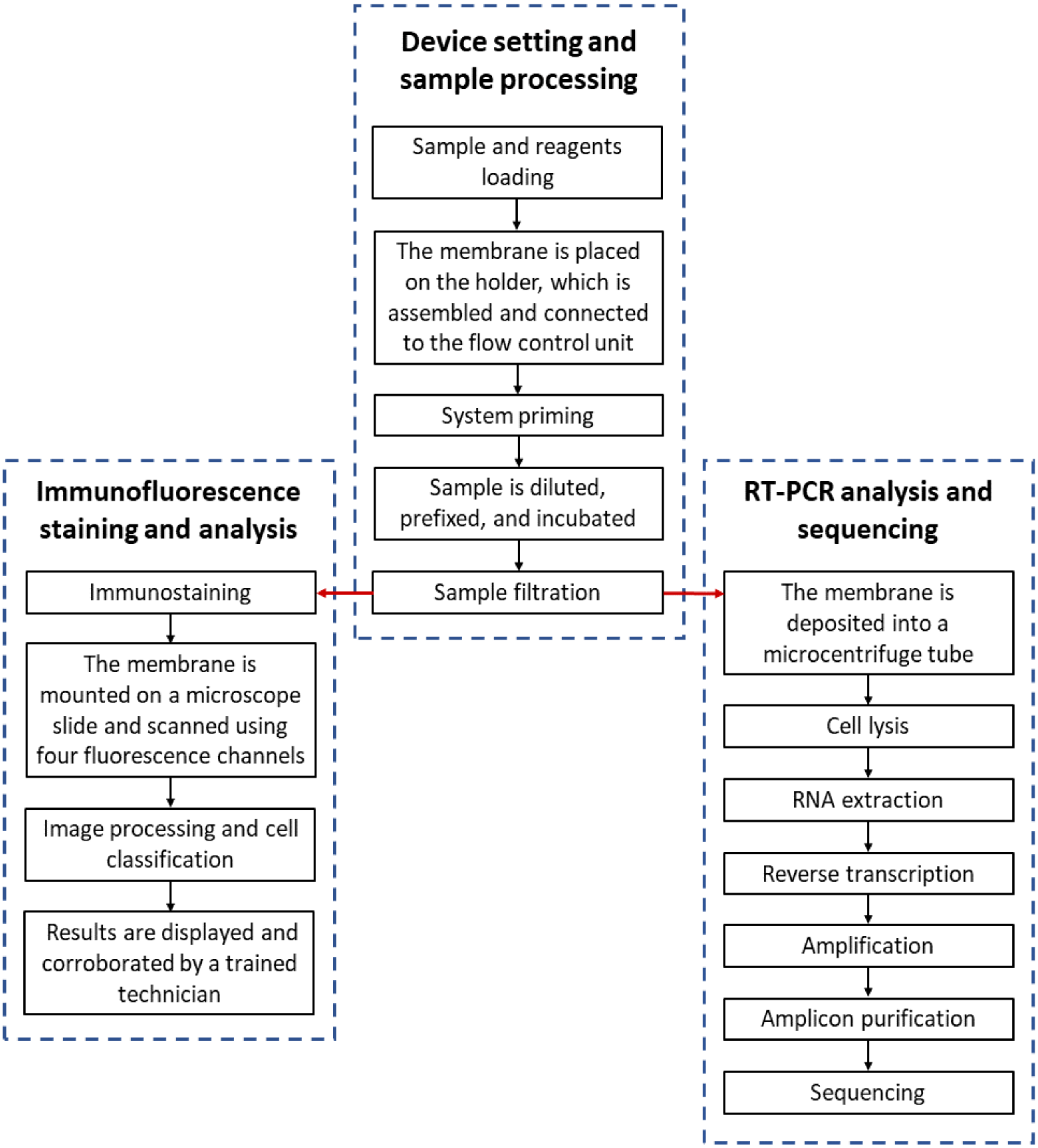

Supplementary Fig. S2 – To assess the clonogenic potential of cancer cells after filtration, PC3- cancer cell suspensions were processed by the device at a flow rate of 2 mL/min. Based on their ability to grow into colonies, it was demonstrated that PC-3 cells maintained their clonogenic potential after filtering. (a) PC-3 cell culture control after 1, 4, and 8 days of incubation. (b) Recovered PC-3 cells after 1, 4, and 8 days of incubation. Scale bar: 100 µm.

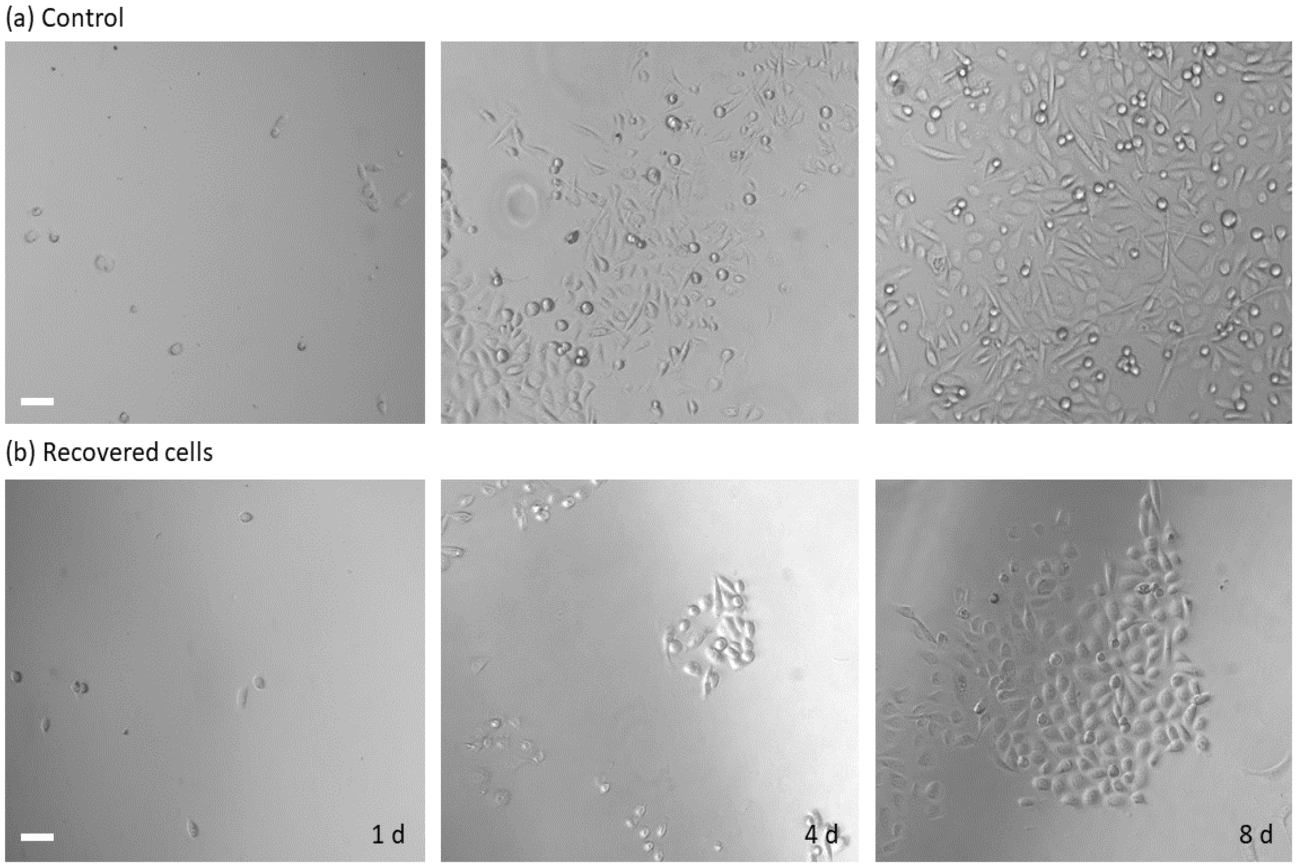

Supplementary Fig. S3 - Uncropped images of the DNA stained gels showed in the main document. (a) RT-PCR analysis of the AR transcript. NTC: Negative control; C+: Positive control; B: Non-spiked blood sample; M: Molecular weight marker; 50: Blood sample spiked with 50 LNCaP cells; 250: Blood sample spiked with 250 LNCaP cells; 500: Blood sample spiked with 500 LNCaP cells; 1000: Blood sample spiked with 1000 LNCaP cells. (b) ACTB was used as a control to assess cDNA synthesis. Gels were viewed using the Midi LED transilluminator (IO Rodeo, Pasadena, CA, USA) and images were taken using a standard cell phone camera, with an exposure time of 330 ms. The saturation of the images in the main document were zeroed using Adobe Photoshop (Adobe Inc., San Jose, CA, USA) without altering the contrast and brightness of the image.

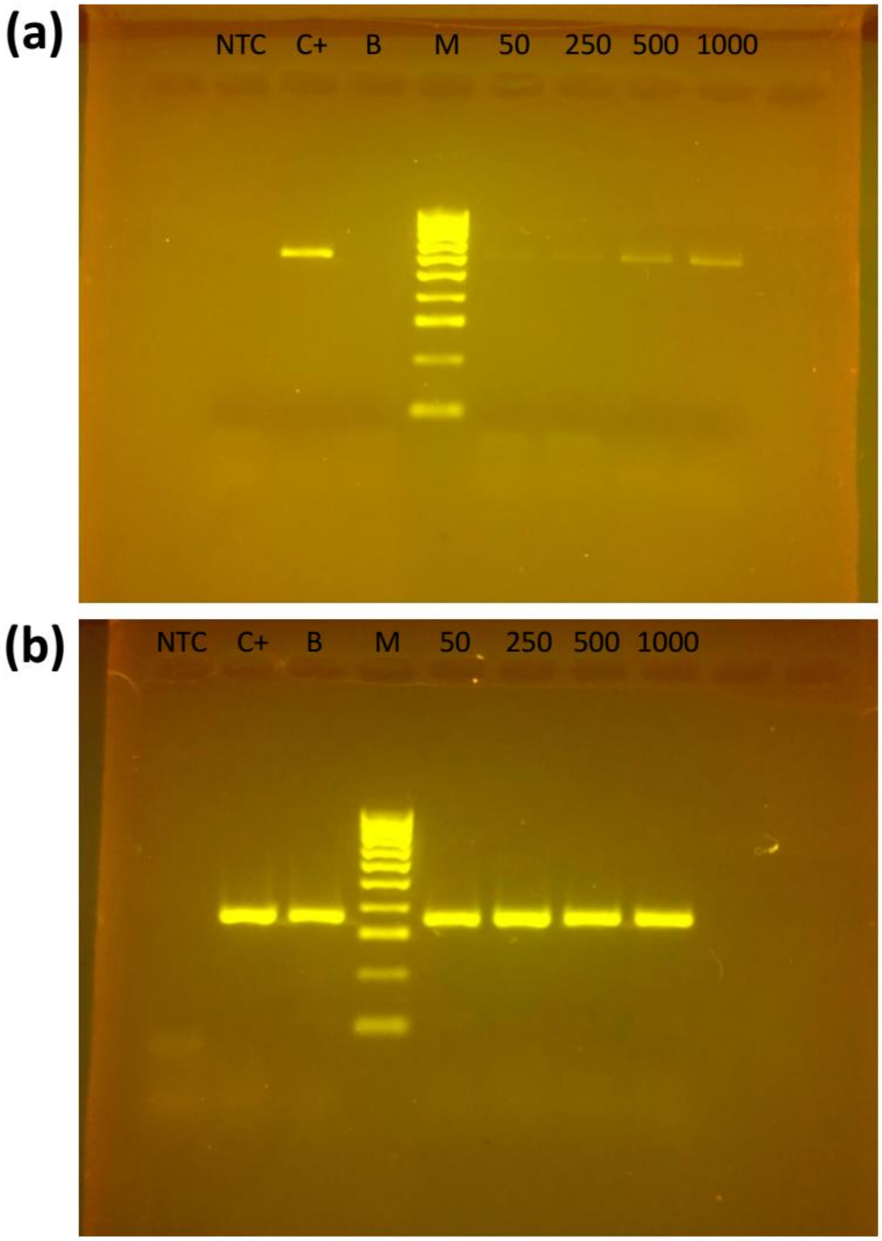

Supplement: Supplementary file 1 — Supplementary Information. [file 41598_2020_63672_MOESM1_ESM.pdf]
